# Comparative Analysis of Mafriwal (*Bos taurus × Bos indicus*) and Kedah Kelantan (*Bos indicus*) Sperm Proteome Identifies Sperm Proteins Potentially Responsible for Higher Fertility in a Tropical Climate

**DOI:** 10.3390/ijms140815860

**Published:** 2013-07-30

**Authors:** Ali Ashrafzadeh, Sheila Nathan, Saiful Anuar Karsani

**Affiliations:** 1 School of Biosciences and Biotechnology, Faculty of Science and Technology, Universiti Kebangsaan Malaysia, Bangi UKM 43600, Selangor, Malaysia; E-Mails: ali_ash_3000@yahoo.com (A.A.); sheila@ukm.my (S.N.); 2 Institute of Biological Sciences, Faculty of Science, University of Malaya, Kuala Lumpur 50603, Malaysia; 3 University of Malaya Centre for Proteomics Research, University of Malaya, Kuala Lumpur 50603, Malaysia

**Keywords:** sperm, sperm proteins, fertility, motility, 2D PAGE

## Abstract

The fertility of zebu cattle (*Bos indicus*) is higher than that of the European purebred (*Bos taurus*) and crossbred (*Bos taurus × Bos indicus*) cattle in tropical areas. To identify proteins related to the higher thermo-tolerance and fertility of Zebu cattle, this study was undertaken to identify differences in sperm proteome between the high fertile Malaysian indigenous zebu cattle (Kedah Kelantan) and the sub-fertile crossbred cattle (Mafriwal). Frozen semen from three high performance bulls from each breed were processed to obtain live and pure sperm. Sperm proteins were then extracted, and two-dimensional gel electrophoresis performed to compare proteome profiles. Gel image analysis identified protein spots of interest which were then identified by liquid chromatography mass spectrometry quadrupole time-of-flight (LC MS/MS Q-TOF). STRING network analysis predicted interactions between at least 20 of the identified proteins. Among the identified proteins, a number of motility and energy related proteins were present in greater abundance in Kedah Kelantan. Sperm motility evaluation by Computer Assisted Semen Analysis (CASA) confirmed significantly higher motility in Kedah Kelantan. While results from this study do identify proteins that may be responsible for the higher fertility of Kedah Kelantan, functional characterization of these proteins is warranted to reinforce our understanding of their roles in sperm fertility.

## 1. Introduction

Due to the low production performance of local cattle breeds, Malaysia is still reliant on importation for up to 80% of the nation’s beef and milk consumption [1,2]. In attempting to resolve this problem, a tropicalized breed, Mafriwal (M), with higher production potential was developed by crossbreeding between Sahiwal (*Bos indicus*) and Friesian (*Bos taurus*) cattle [3]. Mafriwal showed higher milk production and weight gain compared to indigenous breeds for example, Kedah Kelantan (KK). However, its fertility was shown to suffer from heat stress [3,4].

Heat stress has been shown to compromise animal welfare [5] as well as weight gain, milk production, and female and male fertility [6–8]. Scrotal insulation in Holstein-Friesian and Belgian Blue bulls (*Bos taurus*) has been shown to increase flagella defects and reduced the percentage of motile sperm [9]. Similarly, as a result of an increase in scrotal temperature in Nelore cattle (*Bos taurus × Bos indicus*), sperm nuclear integrity and the rate of blastocyst formation in IVF were decreased [10].

It has been shown that heat stress damaged the developing spermatozoa in mice, but heat tolerance can be inherited to the progenies [11]. During evolutionary separation from European cattle (*Bos taurus*), Zebu cattle (*Bos indicus*) have acquired genes that confer thermo-tolerance at the cellular and physiological levels. Compared to European breeds, the tissues’ resistance to heat flow, from the core of the body to the skin, is lower in Zebu cattle while sweat glands are larger. Properties of hair coat in Zebu cattle improved conductive and convective heat loss. It also reduced absorption of solar energy through radiation [12]. The higher efficiency of thermoregulation in *Bos indicus* testis compared to the crossbreed (*Bos taurus × Bos indicus*) and *Bos taurus* bulls is due to differences in testicular vascular cone and testicular morphology. Lower testicular temperature has been shown to be associated with higher semen quality and sperm production in *Bos indicus* bulls under heat stress [13].

Thus, in an attempt to identify proteins related to higher thermo-tolerance and fertility, we used proteomics to compare the sperm proteome of the high fertile Malaysian indigenous zebu cattle (Kedah Kelantan) and the sub-fertile crossbred cattle (Mafriwal). These identified proteins may potentially be developed into fertility markers and will provide us with an understanding of which proteins are associated with higher fertility in tropical climates.

## 2. Results and Discussion

### 2.1. 2DGE, Protein Identification by LC MS/MS Q-TOF and GO Analysis

**Figure 1:**
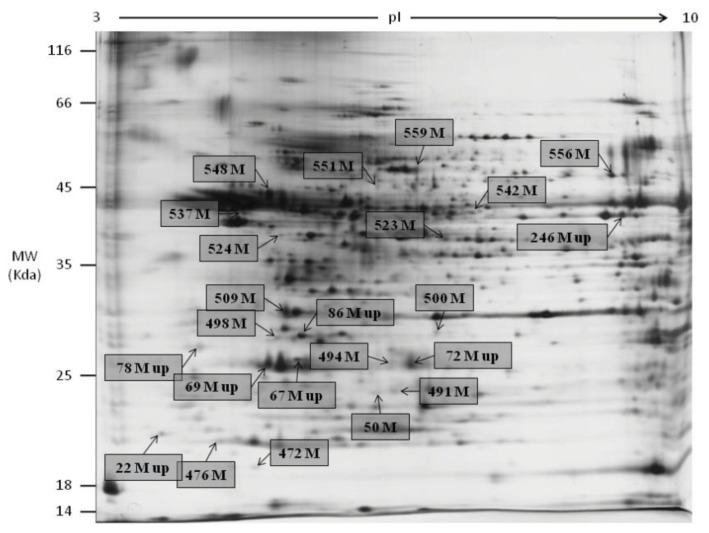
Representative gel showing protein spots with different abundance in Mafriwal sperm.

**Figure 2:**
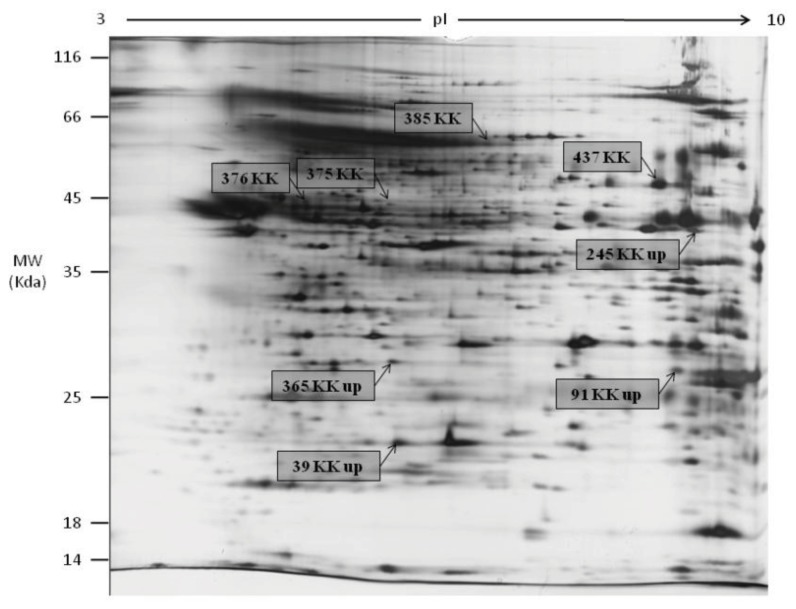
Representative gel showing protein spots with different abundance in Kedah Kelantan sperm.

**Figure 3:**
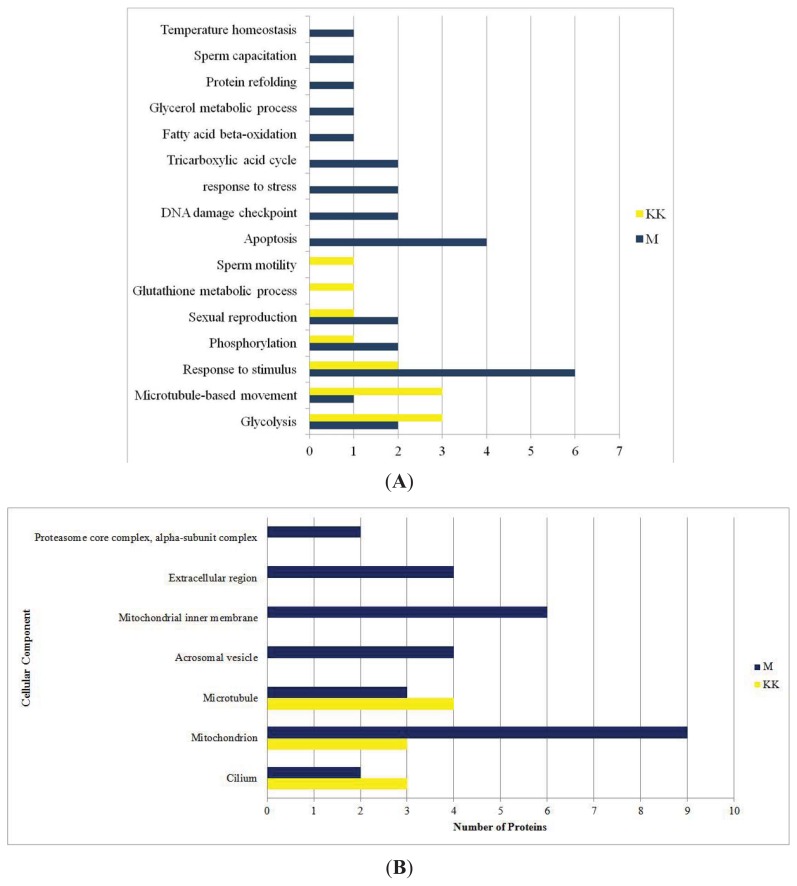
As a result of Gene Ontology (GO) analysis, regulated proteins from Kedah Kelantan (KK) and Mafriwal (M) sperm were classified based on their role in biological processes (**A**) and their location in the cell (**B**).

2DGE was used to resolve and identify potential fertility and environmental compatibility protein marker(s) in KK (Kedah Kelantan, indigenous high fertile Malaysian breed) and M (Mafriwal, sub-fertile crossbred cattle) sperm. Image analysis of silver stained gels showed that 627 individual protein spots were resolved (Figure 1—representative gel for M; Figure 2—representative gel for KK). All gels were highly reproducible, with clearly defined spots. Comparative quantitative analysis of the 2DGE gels revealed that 60 protein spots were significantly different (99% confidence level) in their abundance within the two samples. Only protein spots with a fold change of at least 1.5× were considered as being differentially expressed. A total of 44 protein spots were unambiguously identified by tandem mass spectrometry. Their identities are shown in Table 1. Spot numbers in Figures 1 and 2 refer to the Spot ID in Table 1. These proteins can be classified under different categories based on their function in biological processes (Figure 3A), and cellular components (Figure 3B).

### 2.2. Protein Network Analysis

**Figure 4:**
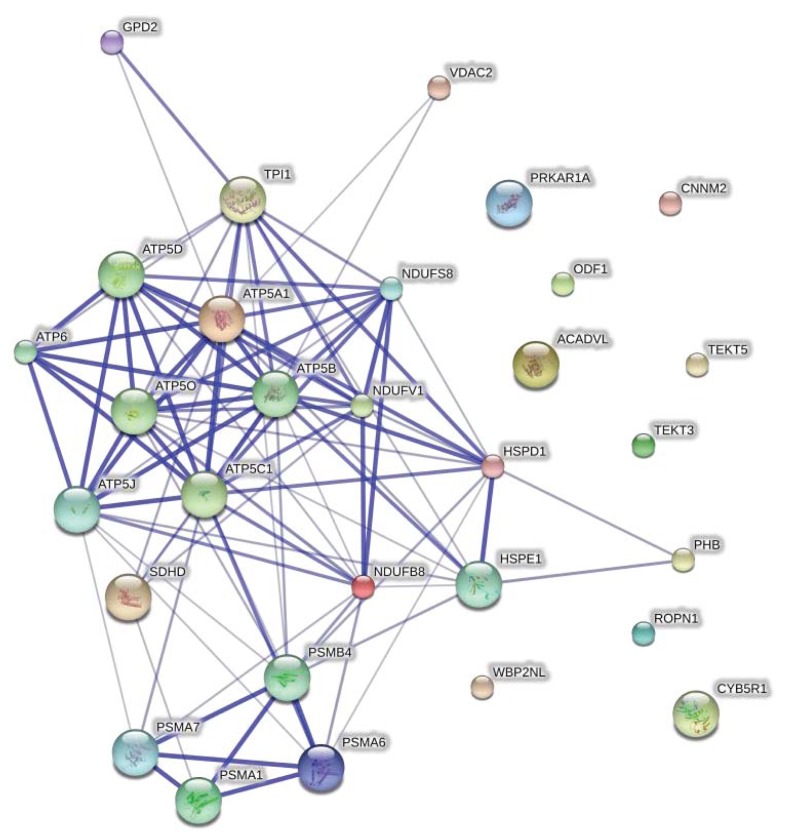
STRING interaction network showing the association between differentially expressed proteins. The interaction map was generated using default settings (Medium confidence of 0.4 and 7 criteria for linkage: neighbourhood, gene fusion, co-occurrence, co-expression, experimental evidences, existing databases and text mining). Ten additional interplay proteins were also added to each network. The protein names and gene symbols used in this network are listed in Table 2.

STRING network analysis of protein-protein interactions was performed to identify functionally linked proteins and determine the potential biological processes affected [14]. The network is presented under confidence view, whereby stronger associations are represented by thicker lines or edges and vice versa. Proteins are represented as nodes. Ten additional interacting proteins were added to provide a more comprehensive view of the interactions. The protein names and gene symbols used in this network are listed in Table 2. All gene symbols were derived from the HUGO Gene Nomenclature Committee (HGNC) [15]. Figure 4 shows the interaction between 21 identified proteins and the 10 additional interactors. Twenty-one proteins were found to be linked either directly or indirectly through one or more interacting proteins, suggesting the existence of known functional linkages.

### 2.3. Proteins with Functions Related to Fertility

Outer dense fibers (ODF) protein was found to be significantly higher in abundance (15.9 fold) in KK and a protein species of kinase anchor protein-4 (AKAP4) was found to be KK specific. As the major proteins in flagella, defects in the ODFs and AKAPs can significantly affect sperm motility [16,17]. ODF2 is an abundant protein in the central scaffold of sperm flagella. Down regulation of ODF2 in globozoospermic sperm showed its modulatory role in sperm motility [18].

A number of proteins that were different in abundance between M and KK were found to exist in multiple forms suggesting the occurrence of post-translational modifications. Three different protein spots were identified as triose phosphate isomerase (TIM). Sperm is translationally and transcriptionally inactive. Therefore, protein post-translational modifications play a crucial role in sperm fertility and hyper-activation. S-nitrosylation and phosphorylation are two major post-translational modifications taking place within the sperm [19,20]. These modifications can activate or deactivate sperm proteins [20,21]. The existence of multiple forms of this protein has been demonstrated before. In a comparison between normozoospermic sperm and asthenozoospermic sperm proteome, Siva *et al.*, identified four different TIM isoforms with different pI values (5.75, 5.95, 6.1 and 6.25) in human spermatozoa, of which the isoform with pI 6.1 was found to be up-regulated in asthenozoospermic sperm with impairment in sperm motility [22].

An important enzyme family involved in detoxification of intermediate metabolites is glutathione-s-transferase (GST). Our results showed GST mu-5 to be higher in KK sperm. GSTs are involved in the conjugation of reactive intermediates with glutathione, thus facilitating their excretion [23]. Sperm motility is closely associated with sperm membrane integrity which is largely affected by reactive oxygen species (ROS) production and its scavenging by antioxidants *i.e.*, glutathione and superoxide dismutase in seminal plasma. ROS affects sperm membrane fluidity by oxidizing polyunsaturated fatty acids. It has been shown that the concentration of antioxidants (glutathione, superoxide dismutase and catalase) in seminal plasma is directly correlated with sperm motility [24].

Succinate dehydrogenase flavoprotein subunit (FP) and 60 KDa heat shock protein were found to be M specific. Heat shock proteins (HSPs) are molecular chaperons as well as being stress induced expressed proteins in different cells [25]. HSP-60 is localized in the mid-piece of ejaculated human sperm [26]. It has been shown that the regulation of different HSPs in human sperm is associated with fertility impairment [22,27]. It has been hypothesized that this protein plays a role in sperm-zona pellucida interaction [28].

We found Izumo4 to be present in greater abundance in M sperm. Izumo is a sperm integral membrane immune-globulin protein family located in the inner acrosomal membrane and equatorial part of sperm plasma membrane [28]. An Izumo gene disrupted mouse line produces normal sperm which can penetrate the zona pellucida, but is incapable of fusing with oocyte [29].

Two different protein spots identified as acrosin binding protein were found to be in higher abundance in M sperm. Acrosin binding protein functions in the packing and storage of acrosin in the acrosome. Although acrosin is one of the major proteolytic enzymes of the acrosome which plays a role in sperm zona pellucida penetration, acrosin-deficient mice remained fertile [30]. This was related to the fact that the acrosome contains several trypsin-like serine proteases that can take over the role of acrosin [31].

Our analysis also showed that a 30 kDa prohibitin was found in lower abundance in KK compared to M sperm. Members of the prohibitin protein family are present in the inner mitochondrial membrane, cytoplasm and nucleus [32,33]. This group of proteins is named after their function which is the prevention of DNA replication. However, it has recently been shown that in some cases they induce cell proliferation and prevent apoptosis [33]. Recently, Wong *et al.* reported a positive correlation between the level of prohibitin and sperm motility by comparing asthenozoospermic and normozoospermic sperm in humans [34].

A number of protein spots were found to be breed-specific. This specificity may be associated with the post-translational modifications of proteins that can change their pI and/or molecular weights. Moreover, the levels of some of these proteins in one breed may be lower than the detectable range of silver staining.

### 2.4. Studies on Sperm Motility

A number of proteins involved in glycolysis were found to be different in abundance between the two samples. Two protein spots identified as pyruvate kinase and three different protein spots identified as triose phosphate isomerase were found to be different in abundance in M and KK. Furthermore, glyceraldehyde dehydrogenase testis specific was missing in M. On the other hand, glycerol-3-phosphate dehydrogenase and very long-chain specific acyl-CoA dehydrogenase that are related to lipid metabolism were found to be M specific. Structural proteins that may be associated with sperm motility were also found to be different in abundance between M and KK sperm. Three different protein spots identified as tubulin (a major component of the flagella) and AKAP4 were found to be KK specific.

Taken together, these results suggested that there existed a difference in the structure of the flagella and in energy utilization between KK and M sperm. These differences may lead to a difference in sperm motility contributing towards a difference in fertility. To investigate this possibility, we performed a sperm motility test by CASA. Our results showed that there was significantly higher sperm motility and flagella lateral amplitude in KK sperm compared to M sperm (Table 3). This supported the observations made in the proteomics analysis with regards to proteins involved in structure and energy utilization in sperm.

## 3. Experimental Section

### 3.1. Semen Sample

Semen from three high performance bulls of Mafriwal (Malaysian synthetic breed, *Bos taurus × Bos indicus*) and Kedah Kelantan (high fertile indigenous breed, *Bos indicus*) were purchased from a cattle breeding center of the National Institute of Veterinary Biodiversity in Malaysia (IBVK) and frozen till used. Prior to use, frozen semen was thawed in a water bath (37 °C for 30 s). Living sperm were separated using BoviPure gradient solution (Nidacon). Briefly, thawed semen was deposited onto the surface of the gradient solution and centrifuged (300× *g*, 20 min at 25 °C) to obtain viable, live sperm.

### 3.2. Protein Extraction

Protein extraction was performed by adding 500 μL of lysis buffer [5 M Urea, 2 M Thiourea, 2% CHAPS, 2% Sulfobetaine 3–10 and 20 mM Dithiothreitol (DTT)] containing a protease inhibitor cocktail (GE Healthcare) to the sperm pellet. Sample homogenization was achieved by freeze-thawing. Extracted samples were centrifuged (16,000× *g*, at 4 °C for 15 min). The supernatant was then collected in an eppendorf tube. Protein quantification was performed using a modified Lowry protein assay kit (BioRad, Philadelphia, PA, USA) [35]. Protein aliquots of extracts (80 μg) were prepared and stored in −80 °C for further use.

### 3.3. Two-Dimensional Gel Electrophoresis (2DGE)

Unless otherwise specified, all reagents and apparatus for protein separation were from GE Healthcare. Experiments were performed as described by the manufacturer with optimizations. Briefly, gel electrophoresis was performed in six replicates for each breed. Non-linear pH gradient IPG strips (24 cm, pH = 3–10) were rehydrated in DeStreak rehydration solution overnight. Rehydrated strips were placed in the IPGphor manifold and 80 μg of protein extract were loaded by cup-loading at the anodic side. Isoelectric focusing was then carried out using an IPGphor III system (GE Healthcare, Uppsala, Sweden), using a five-step program (1 h at 200 V, 1 h at 500 V, 1 h at 1000 V, 3 h gradient to 8000 V and finally on 8000 V to reach a total of 75,000 Vh). After focusing, IPG strips were equilibrated in the first equilibration solution [6 M Urea, 75 mM Tris-Hcl, 29.3% (*v*/*v*) Glycerol, 2% (*w*/*v*) SDS, 0.002% (*w*/*v*) Bromophenol blue and 100 mg DTT per 10 mL of final solution] for 15 min. The strips were transferred to the second equilibration buffer (with the same composition as the first solution except DDT, which was replaced by 250 mg of Iodoacetamide per 10 mL of final solution) and shaken for another 15 min. Strips were then transferred to the second dimension SDS-PAGE (10%) and sealed with agarose sealing solution [0.5% agarose, 0.002% (*w*/*v*) Bromophenol blue]. Second dimension electrophoresis was carried out on an Ettan DALT Six system, using a two stage program (1 h, 10 mA/gel, 80 V and 40 mA/gel, 500 V until the dye reached the bottom of the gel).

Following SDS-PAGE, protein spots were visualized using protocols described in the PlusOne™ Silver staining kit. The complete protocol was followed for analytical gels. For preparative gels, the protocol was modified so that glutaraldehyde was omitted from the sensitization step and formaldehyde omitted from the silver reaction step [36]. Gel images were scanned using a densitometer and image analysis was performed using the ImageMaster 2D Platinum 7.0 using parameters previously described [37,38].

### 3.4. LC-MS/MS Q-TOF Protein Identification

#### 3.4.1. In-Gel Tryptic Digestion

Protein spots were cut out from preparative gels (gel plugs). Destaining was then performed by immersing the gel plugs in destaining solution (15 mM Potassium Ferricyanide in 50 mM Sodium Thiosulphate) for 15 min with regular shaking. Subsequently, the proteins were reduced (10 mM DDT in 100 mM Ammonium Bicarbonate, 30 min at 60 °C) and alkylated (55 mM Iodoacetamide in 100 mM Ammonium Bicarbonate, incubated in dark for 20 min). The gel plugs were then dehydrated in 50% Acetonitrile in 100 mM Ammonium Bicarbonate followed by 100% Acetonitrile. Tryptic digestion was performed overnight at 37 °C using Trypsin Gold Mass Spectrometry grade (Promega, Sunnyvale, CA, USA). Following tryptic digest, samples were cleaned up using Zip Tip (Millipore, Billerica, MA, USA) according to the manufacturer’s instructions. The samples were then dried using a vacuum concentrator and stored at −20 °C until used.

#### 3.4.2. Mass Spectrometry

All instruments and software used for mass spectrometry analysis were from Agilent (Agilent, Santa Clara, CA, USA) unless otherwise stated.

Digested samples were reconstituted in 5 μL of the initial LC mobile phase (0.1% formic acid). Peptide separation was performed by Nano-flow LC 1200 series (Agilent, Santa Clara, CA, USA) directly connected to Accurate-Mass Q-TOF 6520 with an ESI ion source. Digested peptides were first enriched using an enrichment column. Peptides were then separated on an HPLC Chip-Protein column (C18 reverse phase, 300 Å, 43 mm, Agilent, Santa Clara, CA, USA) with a 3%–50% linear gradient of solvent B (90% Acetonitrile and 0.1% Formic acid) for 30 min with a flow rate of 0.3 μL/min. Mass spectra were acquired using MassHunter Qual acquisition software (Agilent, Santa Clara, CA, USA). Each mass spectra acquisition (8 masses per second from 115 to 3000 m/z) was followed by collision-induced dissociation of the four most intensive ions. MS/MS data were acquired in the range of 50–3000 *m*/*z*.

#### 3.4.3. Database Searching

Spectrum Mill software (Agilent, Santa Clara, CA, USA) was set to search MS/MS acquired data against Swiss-Prot mammalian database, assuming tryptic digestion. Mass-tolerance of precursor and product ions was set to ±20 and ±50 ppm, respectively. Due to the use of iodoacetamide for alkylation in sample preparation, carbamidomethylation was specified as a fixed modification and oxidized methionine as a variable modification. Precursor mass shift was specified to −18 and +177 Da.

Identified proteins and peptides were validated using the Spectrum Mill software based on the software default settings. Protein score specified to be more than 20, peptide mass error less than 5 ppm, forward-reverse score more than 2, peptide score more than 6 and Scored Peak Intensity (%SPI) more than 60 percent. Validated proteins that shared at least two peptides were assigned to the same group. Additionally, identification was performed by PEAKS software (Bioinformatics solutions, Waterloo, ON, Canada) for peptide *de novo* sequencing, identifying post-translational modifications, and mutations. For *de novo* sequencing, total local confidence (TLC) was set to 3 and average local confidence (ALC) to 50%. Identified proteins were filtered by false discovery rate (FDR, <5%) for peptide-spectrum matches.

#### 3.4.4. Bioinformatics Analysis

Blast2Go, an online tool for gene ontology analysis, was used to identify annotations of our protein data sets and group them based on their function in biological processes and cellular components [39]. Categorization of functional and sub-cellular distribution of proteins was performed based on a Swiss-Prot/TrEMBL database search. Protein-protein interactions were predicted using Search Tool for the Retrieval of Interacting Genes/Proteins (STRING) database v9.05 [40]. The Swiss-Prot identifier for the genes (e.g., ENOA_HUMAN for alpha-enolase), in ‘protein mode’, was used to search against the STRING database. Network analysis was set at medium stringency (STRING score = 0.4). Proteins were linked based on seven criteria; neighbourhood, gene fusion, cooccurrence, co-expression, experimental evidences, existing databases and text mining.

#### 3.4.5. Sperm Motility Test by Computer Assisted Semen Analysis (CASA) System

Sperm motility was analyzed using a sperm analyzer (CASA-system, HTM-IVOS, Version 10, Hamilton-Thorne Biosciences, Beverly, MA, USA). CASA slides were first warmed to 37 °C. Two 10 μL fractions of washed sperm were introduced into two chambers of the CASA slide. The following eight parameters were evaluated: percentage of motile sperm, percentage of sperm with progressive motility, percentage of sperm with rapid motility, path velocity (μm/s), progressive velocity (μm/s), track speed (μm/s), flagella lateral amplitude (μm) and beat frequency (Hz). The manufacturer’s settings were adjusted to obtain clear identification of individual spermatozoa. Three slides per breed and five randomly selected slide fields per slide were scanned. After every set of scans, playback mode was used to analyze the video sequences of the last slide field in order to validate the sperm and its trajectory detection. The student t-test was performed on the final results using the SPSS software (IBM SPSS statistics 21. IBM Corporation, Armonk, NY, USA).

## 4. Conclusions

We have successfully performed a proteomic analysis comparing sperm from KK and M. A number of proteins known to be associated with sperm fertility and motility were found to be present at different abundance within the two samples. A significant difference in sperm motility between the two breeds was confirmed by CASA analysis. With proper validation, these proteins may be developed into fertility biomarkers.

## Figures and Tables

**Table 1:** Proteins identified using LC MS/MS Q-TOF.

Ascension number	Full name	Species	Score	Coverage (%) (number of peptides)	Remarkes	Fold change	Spot ID
Q2TA16	Coiled-coil domain-containing protein 65	*Bos taurus*	73.7	21 (13)	KK specific	-	375
O35774	A-kinase anchor protein 4	*Rattus norvegicus*	110.8	17 (16)	KK specific isoform	-	385
Q2KJE5	Glyceraldehyde-3-phosphate dehydrogenase, testis-specific	*Bos taurus*	76.8	14 (5)	KK specific isoform	-	437
P81947	Tubulin alpha-1B chain	*Bos taurus*	17.8	6 (2)	KK specific isoform	-	376
Q2HJ86	Tubulin alpha-1D chain	*Bos taurus*	17.8	6 (2)	KK specific isoform	-	376
Q32KN8	Tubulin alpha-3 chain	*Bos taurus*	73.3	10 (5)	KK specific isoform	-	375
Q32KN8	Tubulin alpha-3 chain	*Bos taurus*	74.0	15 (6)	KK specific isoform	-	376
P11979	Pyruvate kinase isozymes M1/M2	*Felis catus*	84.2	11 (6)	KK specific isoform	-	437
Q9MZ13	Voltage-dependent anion-selective channel protein 3	*Bos taurus*	87.5	16 (5)	KK up regulated	15.9	91
Q9Z1B2	Glutathione S-transferase Mu 5	*Rattus norvegicus*	139.6	61 (17)	KK up regulated	2.0	39
Q3MHW9	NADH-cytochrome b5 reductase 1	*Bos taurus*	45.2	9 (3)	KK up regulated	15.9	91
Q29438	Outer dense fiber Protein 1	*Bos taurus*	168.2	44 (20)	KK up regulated	15.9	91
Q29438	Outer dense fiber protein 1	*Bos taurus*	93.0	22 (6)	KK up regulated	2.3	365
Q5E956	Triose phosphate isomerase	*Bos taurus*	119.6	71 (14)	KK up regulated	2.3	365
P53353	Sperm acrosomal protein FSA-ACR.1 (Fragment)	*Vulpes vulpes*	54.2	4 (2)	M specific	-	472
P53353	Sperm acrosomal protein FSA-ACR.1 (Fragment)	*Vulpes vulpes*	48.3	9 (3)	M specific	-	476
Q3MHM5	Tubulin beta-4B chain	*Bos taurus*	45.3	5 (2)	M specific	-	537
P48818	Very long-chain specific acyl-CoA dehydrogenase, mitochondrial	*Bos taurus*	66.1	6 (4)	M specific	-	551
P26436	Acrosomal protein SP-10	*Homo sapiens*	54.2	6 (2)	M specific	-	472
A6QLU1	Glycerol-3-phosphate dehydrogenase, mitochondrial	*Bos taurus*	103.0	24 (19)	M specific	-	559
A3KFF6	Postacrosomal sheath WW domain-binding protein	*Bos taurus*	94.3	25 (7)	M specific	-	509
Q3T0X5	Proteasome subunit alpha type-1	*Bos taurus*	30.3	7 (2)	M specific	-	494
Q2YDE4	Proteasome subunit alpha type-6	*Bos taurus*	49.7	20 (5)	M specific	-	491
A6H782	Tektin-3	*Bos taurus*	120.7	31 (16)	M specific	-	542
Q1ZYL8	Izumo sperm-egg fusion protein 4	*Homo sapiens*	40.5	6 (2)	M specific isoform	-	491
P31081	60 kDa heat shock protein, mitochondrial	*Bos taurus*	104.8	19 (12)	M specific isoform	-	548
P00514	cAMP-dependent protein kinase type I-alpha regulatory subunit	*Bos taurus*	72.3	12 (5)	M specific isoform	-	524
P10173	Fumarate hydratase, mitochondrial	*Sus scrofa*	88.0	21 (10)	M specific isoform	-	523
P11979	Pyruvate kinase isozymes M1/M2	*Felis catus*	150.2	32 (23)	M specific isoform	-	556
Q3T064	Ropporin-1	*Bos taurus*	49.4	8 (2)	M specific isoform	-	476
P31039	Succinate dehydrogenase [ubiquinone] flavoprotein subunit, mitochondrial	*Bos taurus*	93.4	14 (10)	M specific isoform	-	559
Q5E956	Triose phosphate isomerase	*Bos taurus*	80.0	24 (6)	M specific isoform	-	498
P68002	Voltage-dependent anion-selective channel protein 2	*Bos taurus*	120.7	21 (5)	M specific isoform	-	500
Q1ZYL8	Izumo sperm-egg fusion protein 4	*Homo sapiens*	48.3	6 (2)	M up regulated	2.0	72
P53353	Sperm acrosomal protein FSA-ACR.1 (Fragment)	*Vulpes vulpes*	82.6	11 (5)	M up regulated	3.2	78
Q8NEB7	Acrosin-binding protein	*Homo sapiens*	41.6	4 (2)	M up regulated	6.5	50
Q29016	Acrosin-binding protein (Fragment)	*Sus scrofa*	81.2	6 (4)	M up regulated	3.8	69
P19483	ATP synthase subunit alpha, mitochondrial	*Bos taurus*	169.9	36 (23)	M up regulated	3.4	246
Q3T165	Prohibitin	*Bos taurus*	135.4	40 (12)	M up regulated	1.5	67
Q3T165	Prohibitin	*Bos taurus*	98.1	31 (9)	M up regulated	3.8	69
Q3T064	Ropporin-1	*Bos taurus*	48.9	8 (2)	M up regulated	4.3	22
Q2YDI7	Tektin-5	*Bos taurus*	168.0	47 (27)	M up regulated	3.4	246
Q5E956	Triosephosphate isomerase	*Bos taurus*	136.4	54 (14)	M up regulated	1.9	86

**Table 2:** Protein names and abbreviations used for STRING network analysis.

Abbreviation	Protein name
**Proteins Subjected to STRING Analysis:**	
NDUFB8	NADH dehydrogenase [ubiquinone] 1 beta subcomplex subunit 8
ATP5A1	ATP synthase subunit alpha
ACADVL	Very long-chain specific acyl-CoA dehydrogenase
ODF1	Outer dense fiber protein 1
TEKT3	Tektin-3
PSMA1	Proteasome subunit alpha type-1 (EC 3.4.25.1)
ROPN1	Ropporin-1 (Rhophilin-associated protein 1)
PRKAR1A	cAMP-dependent protein kinase type I-alpha regulatory subunit
PSMA6	Proteasome subunit alpha type-6 (EC 3.4.25.1)
GPD2	Glycerol-3-phosphate dehydrogenase, mitochondrial Precursor (GPDH-M)(GPD-M)(EC 1.1.5.3)
HSPD1	60 kDa heat shock protein, mitochondrial Precursor (Heat shock protein 60)(HSP-60)
CNNM2	cyclin M2
VDAC2	Voltage-dependent anion-selective channel protein 2 (VDAC-2) (Outer mitochondrial membrane protein porin 2)
WBP2NL	Postacrosomal sheath WW domain-binding protein (WW domain-binding protein 2-like)
SDHD	Succinate dehydrogenase [ubiquinone] cytochrome b small subunit
TEKT5	Tektin-5
PHB	Prohibitin
TPI1	Triosephosphate isomerase (TIM)(EC 5.3.1.1)
CYB5R1	NADH-cytochrome b5 reductase 1 (b5R.1)(EC 1.6.2.2)
NDUFV1	NADH dehydrogenase [ubiquinone] flavoprotein 1, mitochondrial Precursor (EC 1.6.5.3)
**Predicted Functional Partners:**	
ATP5C1	ATP synthase subunit gamma
ATP5O	ATP synthase subunit O
ATP5D	ATP synthase subunit delta
ATP5B	ATP synthase subunit beta (EC 3.6.3.14);
ATP6	ATP synthase subunit a
PSMB4	Proteasome subunit beta type-4 Precursor (EC 3.4.25.1)
HSPE1	10 kDa heat shock protein, mitochondrial (Hsp10)
ATP5J	ATP synthase-coupling factor 6
NDUFS8	NADH dehydrogenase [ubiquinone] iron-sulfur protein 8 (EC 1.6.5.3)
PSMA7	Proteasome subunit alpha type-7 (EC 3.4.25.1)

**Table 3:** Sperm motility analysis by CASA.

Motile Sperm (%)			

Breed	Mean	Standard	*p* value
KK	82	1.7321	0.018 *
Mafriwal	72.667	3.7859	

**Progressive (%)**			

**Breed**	**Mean**	**Standard**	***p*value**

KK	51.333	3.7859	0.086
Mafriwal	44.333	3.7859	

**Rapid Cells (%)**			

**Breed**	**Mean**	**Standard**	***p*value**

KK	69.667	1.1547	0.002 **
Mafriwal	57	2.6458	

**Path Velocity (μm/s)**			

**Breed**	**Mean**	**Standard**	***p*value**

KK	130.733	3.72	0.062
Mafriwal	120.367	5.9	

**Progressive Velocity (μm/s)**			

**Breed**	**Mean**	**Standard**	***p*value**

KK	111.267	4.9743	0.272
Mafriwal	105.767	5.6012	

**Track Speed (μm/s)**			

**Breed**	**Mean**	**Standard**	***p*value**

KK	206.767	8.5582	0.018 *
Mafriwal	178.667	9.2511	

**Lateral Amplitude (μm)**			

**Breed**	**Mean**	**Standard**	***p*value**

KK	8.067	0.8622	0.048 *
Mafriwal	6.6	0.2646	

**Beat Frequency**			

**Breed**	**Mean**	**Standard**	***p*value**

KK	26.9	2.1703	0.279
Mafriwal	28.733	0.5033	

**Straightness (%)**			

**Breed Mean Standard*p*value**			

KK	84.333	1.5275	0.349
Mafriwal	85.333	0.5774	

* Statistically significant over 95% confidence level;

** Statistically significant over 99% confidence level.
